# Mattering and Self-Esteem as Bulwarks Against the Consequences of Financial Strain for Loneliness in Later Life: Differentiating Between- and Within-Person Processes

**DOI:** 10.1177/01640275231221326

**Published:** 2023-12-25

**Authors:** Alex Bierman, Laura Upenieks, Yeonjung Lee, Fahimeh Mehrabi

**Affiliations:** 1 Department of Sociology, 2129 University of Calgary, Calgary, AB, Canada; 2 Department of Sociology, 14643 Baylor University, Waco, TX, USA; 3 School of Social Welfare, 26729 Chung-Ang University, Seoul, Korea; 4 Faculty of Social Work, 2129 University of Calgary, Calgary, AB, Canada

**Keywords:** loneliness, financial strain, mattering, self-esteem

## Abstract

Financial strain likely constitutes a principal risk for loneliness in later-life, but a strong sense of mattering and self-esteem may mitigate these consequences by both offsetting and buffering the influence of financial strain. We test these arguments using data from a national longitudinal survey of older adults gathered between 2021 and 2022 (*N* = 2384), as nations emerged from the COVID-19 pandemic. Application of a within-between modelling strategy facilitates differentiation of inter-individual (i.e., between-person) and intra-individual (i.e., within-person) factors. Between-person financial strain is associated with greater loneliness, but within- and between-person mattering and self-esteem offset this association by forestalling loneliness. Between-person mattering buffers between-person financial strain, but between-person self-esteem buffers within-person financial strain. Consequently, within-person financial strain is associated with greater loneliness only at low levels of between-person self-esteem. In summary, accruing a strong sense of worth contributes to protecting older adults from the adverse consequences of financial strain for loneliness.

The study of loneliness is a critical topic in research on aging. Loneliness is “a subjective negative experience that results from inadequate meaningful connections” (Fried et al., 2020. p. 114). Social isolation and the quality of one’s relationships may constitute antecedent causes of loneliness (Hawkley et al., 2022), but loneliness is distinct by representing individual experiences of one’s relationships (Prohaska et al., 2020). Loneliness is especially relevant to the study of aging because loneliness increases with age in later-life (Cohen-Mansfield et al., 2016; Hawkley et al., 2022), and these age-related increases have grown stronger over time (Newmyer et al., 2022). The increase in loneliness that accompanies aging is a substantial threat to the well-being of older adults because loneliness is deleteriously associated with a broad set of health outcomes, including cognition, depression, disability, cardiovascular disease, and mortality (Berg-Weger & Morley, 2020). As the proportion of older adults in North America is expected to grow rapidly (Denton & Spencer, 2019; Preston & Vierboom, 2021), identifying the factors that lead to loneliness—and the resources that can be utilized to prevent these effects—is a central mission for ensuring population health.

In the current research, we apply a stress process perspective to the study of loneliness among older adults. The stress process perspective is a paradigmatic theoretical perspective in the sociological study of mental health which positions financial strain as paramount among social stressors (Kahn & Pearlin, 2006; Pearlin & Bierman, 2013). Financial strain denotes everyday lived experiences of difficulty affording basic needs and making ends meet (Upenieks & Ellison, 2022, with the prominence of financial strain as a social stressor due to the ample psychological abrasion caused by an inability to provide for one’s self (Pearlin, 1999). Recent research supports the stress process perspective’s emphasis on financial strain by showing that financial stress was preponderant in potency among stressors experienced during the COVID-19 pandemic (Qian & Fan, 2023). Although financial strain has been examined as a source of psychological distress (Guan et al., 2022), we argue in this paper that struggles to afford daily life will also lead to loneliness by degrading social relationships and alienating individuals from others. Moreover, since financial strain tends to increase across later-life (Bierman, 2014), financial strain is of central interest as a factor that may contribute to greater loneliness as individuals age.

Yet, a stress process perspective also emphasizes contingencies in the consequences of stress exposure (Pearlin & Bierman, 2013). A primary tenet of this perspective is that components of the self-concept are key psychological resources that have salubrious effects on individual well-being and also weaken the deleterious effects of stressors (Pearlin, 1999; Thoits, 2013). Self-esteem and mattering are two components of the self-concept that are particularly critical in the study of the stress process (Fazio, 2010), with sociological interest in these components guided by their shared origins in social relationships and structures of social stratification (Bonhag & Froese, 2022; Keith & Thompson, 2019; Serpe et al., 2019). Mattering and self-esteem each represents a type of self-assessed worth—self-esteem referring to a sense of self-worth and mattering a sense of social worth (Bonhag & Froese, 2022; Orth & Robins, 2014). In this paper, we argue that mattering and self-esteem are likely to negate the effects of financial strain on loneliness in two ways. First, through direct associations that mitigate loneliness, which will serve to offset the degree to which financial strain increases loneliness. Second, by weakening the consequences of financial strain for loneliness in a process of “stress buffering” (Louie & Upenieks, 2022). However, the mutual social origins of mattering and self-esteem, together with their shared focus on a sense of worth, suggest that these two components of the self-concept should be considered in conjunction to avoid spuriously attributing the effects of one to the other.

In this study, we analyze a national longitudinal survey of Canadian older adults gathered between 2021 and 2022 during the COVID-19 pandemic. We first examine how financial strain is associated with loneliness, and then how mattering and self-esteem may serve as resources both by offsetting and buffering consequences of financial strain for loneliness. We further elaborate on this nuanced depiction by specifying two distinct levels of these processes. The first refers to aspects of older adults that were stable over the course of the study, and therefore considered “between-person” because the only variation was between individuals (McNeish & Kelley, 2019). Second, we examine “within-person” processes that specifically refer to changes in individuals. We make these distinctions because the between-person analyses demonstrate how stable aspects of financial difficulties and a sense of worth explain the degree to which older adults were consistently lonely during the study. At the same time, the within-person aspect of these analyses shows how changes in financial strain and the self-concept conditioned changes in loneliness, thereby specifying whether increases in mattering and self-esteem in later-life may protect older adults from loneliness.

## Background

In recent years, public health officials worldwide have expressed concern about a “loneliness epidemic,” in light of the pervasiveness of loneliness across the developed world and its debilitating health effects (Berg-Weger & Morley, 2020; Holt-Lunstad et al., 2017). Even prior to the COVID-19 pandemic in Canada, 48% of Canadian adults reported feeling lonely, and almost 2 in 3 wished their friends and family would spend more time with them (McComb et al., 2020). These figures were almost certainly exacerbated as a result of the pandemic. As of the spring of 2021, older adults represented 20% of total COVID-10 cases, 70% of those hospitalized with COVID-19, and 96% of deaths from the virus (Hayden et al., 2023). Because of this initial high fatality among older Canadians, this virus was quickly framed by policymakers and the media as being primarily of concern to “the elderly” (Maclean’s, 2020). While this concern was well-intentioned, some public health units in Canada advised those 65 and over to not leave their homes at all (CBC News, 2020), and many grocery stores implemented “seniors’ hours” so that older people could shop in a safer environment before younger shoppers arrived (Dunham, 2020). Some scholars have suggested such policies targeting older persons increased the likelihood of social isolation of older adults (Monahan et al., 2020).

With a societal backdrop that accentuated the precariousness of loneliness, financial strain was likely to be reinforced as a key determinant of loneliness among older adults through multiple mechanisms. Research suggests that financial strain can cause physical dysregulation that leads to physical and cognitive limitations which may constrain social engagement (Calderón-Larrañaga et al., 2021; Kazlauskaite et al., 2020; Napoleone et al., 2022; Samuel et al., 2019, 2022). Additionally, financial limitations may harm older adults’ abilities to maintain social connections by limiting abilities to freely engage in social activities that necessitate financial expenditures (Corman et al., 2012; Hsu, 2020; Mood & Jonsson, 2016). Financial strain may also degrade the quality of older adults’ social interactions because “[p]eople invest in network relationships—giving gifts and providing favors—and expect future returns from these investments” (Letki & Mieriņa, 2015, p. 218). Financial limitations that inhibit engagement in reciprocation are likely to create awkwardness and hesitancy that curtails personally rewarding and meaningful social interactions (Ferraro & Su, 1999). Further degradations to the quality of social relationships may also emerge because older adults feel shame and embarrassment due to financial problems (Chase & Walker, 2013; Inglis et al., 2019), and are consequently more guarded in social interactions and more reticent in seeking support. Arguments for the adverse effects of financial strain on loneliness are in turn supported by research showing that financial strain is associated with greater loneliness among older adults (Bhatta et al., 2023; Drost et al., 2022; Lee & Bierman, 2019; Victor et al., 2021).

### Mattering and Self-Esteem as Offsetting and Buffering Agents

Guided by a stress process perspective (Pearlin & Bierman, 2013), we place the association between financial strain and loneliness in the context of the self-concept. The self-concept is the summation of thoughts and feelings that are the result of individuals treating themselves as distinct objects of study (Owens & Samblanet, 2013). The self-concept is inherently of sociological interest not only due to its basis in social interactions that are shaped by macro- and meso-social structures (Cast & Stets, 2016; Gecas, 2001), but also because the self-concept can act as a social force with consequences for mental health outcomes (Serpe et al., 2019). The stress process perspective builds from the idea of the self-concept as a social force to undergird a sociological approach to the stress process by focusing on how the self-concept may shape mental health outcomes and the consequences of stress exposure for mental health (Pearlin & Bierman, 2013).

The self-concept is in turn composed of distinct components (Fazio, 2010). One of the most important of these is self-esteem (Zeigler-Hill, 2013). Self-esteem “refers to an individual’s subjective evaluation of his or her worth as a person” (Orth & Robins, 2014, p. 381). We emphasize here that self-esteem refers to the judgment of *individual worth* as a person, which can be differentiated from *social worth* in terms of value to others. This latter aspect of worth is an additional component of the self-concept called “mattering,” which refers to “the personal sense of feeling significant and valued by other people” (Flett, 2022, p. 4). Mattering is therefore reflective of individuals’ evaluations of their own “social significance” (Flett et al., 2012, p. 828). Previous research demonstrates that self-esteem and mattering cohere with a sociological perspective on the structural and interpersonal bases of the self-concept, as social advantages and higher-quality relationships have been shown to build both self-esteem and mattering (Bonhag & Froese, 2022; Schieman & Campbell, 2001; Serpe et al., 2019). Thus, although distinct, self-esteem and mattering frame complimentary aspects of individuals’ self-concept in that both are informed by social structural and interpersonal forces and both refer to an individual’s evaluation of their own worth.

Mattering and self-esteem may crucially intercede in the consequences of financial strain for loneliness in two ways. First, by *offsetting* the consequences of financial strain for loneliness. In this case, mattering and self-esteem will provide a direct negative influence on loneliness, thereby counter-balancing the positive influence of financial strain on loneliness. As Flett and Heisel (2021) suggest, mattering is a psychosocial resource fundamental to the human condition; an older person devoid of mattering to others will lack the basic sense of social significance, connectedness, and acceptance that is needed to flourish. Consequently, older adults lacking in mattering will feel more isolated and be less satisfied with personal relationships. These arguments are supported by research showing that a lack of mattering is associated with greater social isolation and loneliness (Flett et al., 2016; MacDonald et al., 2020; McComb et al., 2020). This research has, however, largely been conducted on samples composed of individuals prior to later-life (see, though, Francis, 2022), thereby leaving open the question of whether the same negative association will be observed among older adults. Self-esteem is also likely to deter loneliness because self-esteem represents the evaluation of one’s individual worth (Orth & Robins, 2014), with the result that self-esteem will be better-equip individuals to avoid loneliness by having the confidence to seek out supportive and meaningful interactions with others (Marshall et al., 2014). Furthermore, although the inverse association between self-esteem and loneliness is supported by previous research among older adults (e.g., Zhao et al., 2017), the dearth of research on mattering and loneliness among older adults also leaves open a question of whether this association will be shown when mattering is taken into account.

A second way that mattering and self-esteem are likely to intercede in the association between financial strain and loneliness is by *buffering* this association, in which mattering and self-esteem weaken the association between financial strain and loneliness. Previous research has demonstrated the role of the self-concept in buffering the consequences of financial strain for health outcomes (Frankham et al., 2020; Koltai et al., 2018), but attention to the role of mattering and self-esteem as buffers in the effects of financial strain on loneliness in later-life is novel. Mattering is likely to act as a buffer, though, by connoting the sense that one “is both valued by other people and who gives value to other people” (McComb et al., 2020, p. 2). Consequently, mattering facilitates a sense that others are receiving rewards from relationships that satisfy reciprocity regardless of financial strain. The sense that others care about one’s well-being is also likely to enhance confidence in interacting with others and reduce anxiety regarding how one is perceived by others (Flett et al., 2016). These effects will facilitate a willingness to be open about financial problems and seek support when confronted by these problems, thereby reinforcing the quality of one’s social relationships and further inhibiting a loss of social interactions. Self-esteem is also likely to act as a buffer by imbuing individuals with greater confidence in managing stressors, thereby leading individuals to see financial strain as less threatening (Shrout & Weigel, 2020). These processes will weaken the stress load caused by financial strain that creates physiological dysregulation and subsequent loss of opportunities for social interactions. Self-esteem is also likely to act as a protective agent because a strong sense of personal worth will prevent feelings of devaluation and stigma (Louie & Upenieks, 2022), thereby halting degradation of social contact and quality of social interactions due to financial hardship.

It is critical to recognize that these offsetting and buffering effects may have been particularly important as the population under study was emerging from the COVID-19 pandemic. Flett and Zangeneh (2020) have argued that everyone needs to feel a sense of significance and importance to others under normal circumstances, but this is especially the case in challenging times and in anxiety-provoking crisis situations that entail separation from others. Thus, individuals’ need to feel that they mattered was likely to be highly salient during the pandemic (Flett & Heisel, 2021), in turn heightening the potency of mattering for directly minimizing loneliness and weakening the influences of potential sources of loneliness. Similarly, a strong sense of self-worth could be vital for stemming fear and anxiety when confronted with the existential threat posed by a pandemic (Lin & Chen, 2021; Rossi et al., 2020). Self-esteem was therefore likely to be prominent as a resource for limiting further diminution of social connection and feelings of alienation in the face of the threat posed by the COVID-19 pandemic, especially when this existential threat was combined with the threat of a lack of ability to provide for basic necessities. We therefore expect that the societal context in which this study was conducted will serve to underscore the offsetting and buffering properties of mattering and self-esteem.

### Differentiating Between- and Within-Person Associations

**Figure 1.**
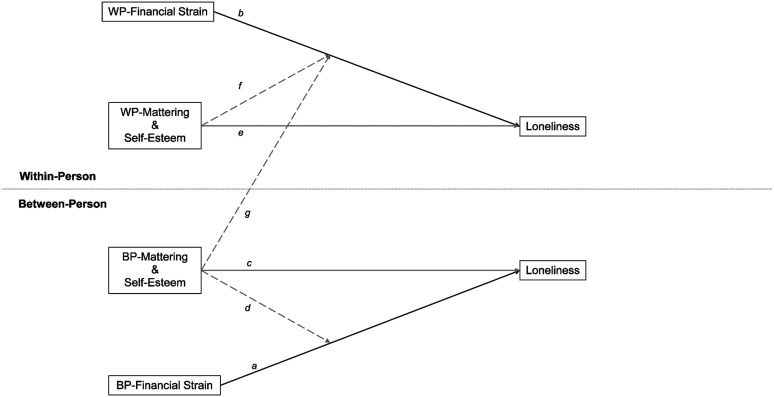
Conceptual model of within-between influences on loneliness. Solid lines show focal hypothesized direct effects. Dashed lines show focal hypothesized buffering effects. Black lines indicate hypothesized positive effects. Grey lines indicate hypothesized negative effects.

It is also critical to attend to between- and within-person differences in these processes because they imply distinct consequences of social inequality for loneliness. To clarify these distinctions, Figure 1 depicts between- and within-person associations in the consequences of financial strain for loneliness and in stress buffering by mattering and self-esteem. Path *a* shows that between-person financial strain leads to greater loneliness. Between-person financial strain in later-life reflects stable interpersonal differences (Hoffman & Stawski, 2009), which tend to amass through the accumulation of advantages and disadvantages across the life-course (Dannefer, 2003; Ferraro & Shippee, 2009; Pudrovska et al., 2005). Consequently, path *a* illustrates that the subjective experience of inadequacy in social relationships is arranged in a hierarchy in later-life based on the accrual of disadvantage across the life-course. Conversely, the intra-individual aspect of financial strain refers to individual variation in financial strain during later-life that may be the result of aging-related transitions (Bierman, 2014), as well as broader economic changes (Bierman, Upenieks, and Lee, 2023). Path *b* therefore shows that individual increases in financial strain contribute to a growth in loneliness in later-life (Cohen-Mansfield et al., 2016; Hawkley et al., 2022). By simultaneously considering the consequences of between- and within-person financial strain for loneliness among older adults, this research can therefore show whether these effects reflect an established hierarchy or a malleable process occurring during later-life.

Research has also shown that between- and within-person aspects of the self-concept can have distinct consequences for the stress process (Koffer et al., 2019). Figure 1 illustrates these multiple roles. First, path *c* illustrates between-person direct associations, in which time-stable aspects of mattering and self-esteem are inversely associated with loneliness, thereby offsetting the positive direct effects of between-person financial strain on loneliness. Second, path *d* shows buffering at the between-person level, in which between-person mattering and self-esteem buffer the association between inter-individual financial strain and loneliness. Focusing on inter-individual associations demonstrates how disadvantages intersect: older adults who not only have more financial strain than others, but also have less self-esteem and mattering, will be especially prone to loneliness.

Paths *e* and *f* illustrate how these between-person associations may be distinct from within-person associations. Path *e* shows that intra-individual increases in self-esteem and mattering abate loneliness, thereby offsetting the ramifications of within-person variation in financial strain for amplifying loneliness. Path *f* shows that intra-individual mattering and self-esteem weaken the association between within-person variation in financial strain and loneliness, thereby providing stress buffering at the within-person level. The intra-individual consequences of mattering and self-esteem show the efficacy of building self-esteem and mattering in later-life, as increases in mattering and self-esteem shield individuals from the consequences of individual escalation in financial strain for loneliness.

Finally, path *g* illustrates a third form of buffering. This is a “cross-level” form of buffering (Snijders & Bosker, 2012), in which between-person aspects of mattering and self-esteem buffer the effects of intra-individual financial strain. Cross-level buffering is distinct from purely between- or within-person buffering by showing that the consequences of within-person increases in financial strain for loneliness will be weaker among older adults who possess stable advantages in self-esteem and mattering.^1^ Thus, we present a nuanced depiction of the relevance of the self-concept for loneliness by clarifying the roles of within- and between-person aspects of mattering and self-esteem in offsetting and buffering the consequences of financial strain.

## Methods

### Data

Data are derived from the Caregiving, Aging, and Financial Experiences (CAFE) study, a national survey intended to examine social conditions and well-being among older Canadians. Data were gathered by the study authors in cooperation with the Angus Reid Forum, a Canadian national survey research firm that maintains an ongoing national panel of Canadian respondents from which nationally representative samples can be drawn. The first wave of the CAFE survey was gathered in late September and early October of 2021 as an online survey conducted among a representative sample of 4010 Canadians between age 65 and 85. The response rate was 56%, but results were statistically weighted according to the most current age, gender, and region Census data to ensure a sample representative of older Canadians.^2^ The follow-up was conducted in late September and early October of 2022, approximately a year later. In total, 2420 respondents were recontacted, for a 60% retention rate. Methods used to address attrition are discussed in the plan of analysis.

### Focal Measures

Focal measures were measured at both baseline and follow-up, with the mean of responses at a specific wave used to measure the construct at that observation point.

*Loneliness* was based on a three-item scale from Hughes and colleagues (2004). Respondents indicated how often in the previous seven days they felt like they lacked companionship, left out, and isolated from other people. Responses were on a scale of 1 (Most or all of the time (5–7 days)) to 4 (Rarely or never (less often than once a day)), with all responses coded so that higher values indicated greater loneliness. A principal components analysis (PCA) conducted on these items indicated one eigenvalue above 1, accounting for 81% of the variance in the items, with a Cronbach’s alpha of .88.

*Financial strain* was measured at baseline using three questions based on previous research by Koltai and colleagues (2018). Respondents indicated how often in the previous three months they had trouble paying the bills, did not have enough money to buy household needs, and how their finances usually worked out by the end of the month. Responses to the first two questions ranged from 1 (Never) to 5 (Very often); responses to the third question ranged from 1 (Not enough to make ends meet) to 5 (A lot of money left over). A principal components analysis of these items at baseline indicated one component with an eigenvalue above 1 that accounted for 77% of the variance in the items, with a Cronbach’s alpha of .85.

*Mattering* was measured using a five-item general mattering scale (Marcus & Rosenberg, 1987). I am important to others; other people pay attention to me; I would be missed if I went away; other people are interested in what I have to say; other people depend on me. For all scales, responses ranged from 1 (Strongly disagree) to 5 (Strongly agree), with all responses coded to denote greater mattering. A principal components analysis of these items at baseline indicated one component with an eigenvalue above 1 that accounted for 58% of the variance in the items, with a Cronbach’s alpha of .81.^3^

*Self-esteem* was measured using a five-item version of the Rosenberg self-esteem scale that has been validated in older adults (Monteiro et al., 2022): At times I think I am no good at all.; all in all, I am inclined to think that I am a failure.; I feel I do have much to be proud of.; on the whole, I am satisfied with myself.; I take a positive attitude toward myself. The response scale and coding were the same as for mattering. A principal components analysis of these items at baseline indicated one component with an eigenvalue above 1 that accounted for 62% of the variance in the items, with a Cronbach’s alpha of .84.

### Covariates

Covariates in statistical analyses focus on background social statuses and social engagement that is likely to predict placement into financial strain and/or loneliness. Time-stable covariates include education, gender, age at baseline, and visible minority status.^4^ Education was measured as a set of dichotomous indicators, in which post-high school (some college/trade school or university), graduated from trade school, and university undergraduate degree or greater were contrasted to a high school degree; less than 3% of the raw sample at baseline had less than a high school degree, and these respondents were included with the comparison category. Age was measured in years. For gender, respondents could identify as a man or woman, but could also choose to self-describe. No respondent chose to self-describe, and gender was therefore coded as a dichotomous variable (0 = men, 1 = women). A common approach to race in Canadian research is a general “visible minority” category (Little, 2016), and in keeping with this approach, visible minority status was a dichotomous variable based on the question, “Would you say you are a member of a visible minority here in Canada (in terms of your ethnicity/race)?” with affirmative answers coded as 1.

Time-varying covariates were measured at both waves. Socioeconomic time-varying covariates include income and retirement status, with time-varying indicators of social engagement including partner status, number of people in the household, frequency of social contact in person, and frequency of social contact online. Income was measured as a set of categories in which less than $25,000 in household income was compared to $25,000 to less than $50,000, $50,000 to less than $100,000, $100,000 to less than $150,000, and $150,000 or more. Because individuals who do not provide income often reside in high income categories and taking non-response into account would contribute to controlling for biases in self-reports, missing income was considered as an additional analytic category. Retirement status was measured as a dichotomous variable in which zero = not retired and 1 = retired. A dichotomous indicator took the values of zero = currently married or living with a romantic partner in a common-law relationship and 1 = not partnered. Number of people in the household was measured on a scale of 1 (respondent lives alone) to 5 (four or more). In-person social contact was measured by asking respondents, “In the past month, how often did you have visits or meetings in-person with any of your friends or members of your family who do not live with you?” Electronic social contact was measured by asking respondents, “In the past month, how often were you in contact with any friends or family who do not live with you through phone calls, texting, or video chat?” Responses to both social contact questions were coded from 1 (Never) to 6 (Once a day or more).

### Methods of Analysis

All analyses were conducted with Stata 18.0 using mixed models, also often referred to as multilevel models, in which variance estimation and associated significance tests take repeated observations of the same individual into account (Snijders & Bosker, 2012).^5^ The primary analyses begin with a mixed model that shows the association between financial strain and loneliness over repeated observations when the time-varying and time-stable aspects of financial strain are not separated. Subsequent models then apply an extension of the mixed model using a “within-between model,” which differentiates intra-individual and inter-individual influences (McNeish & Kelley, 2019). As described in Hoffman and Stawski (2009), in this approach, the mean of financial strain for each respondent (*i*) across time points (*t*) is the person-mean of financial strain (PMFS*i*) and represents the between-person component of financial strain. The person-mean of financial strain is then subtracted from the respondent’s financial strain score at each time point (FS*ti*−PMFS*i*), which represents the within-person component of financial strain at each time-point. Between-person and within-person measures of financial strain are entered into a mixed model as predictors at levels 2 and 1, respectively:

(1) $$Loneliness_{ti}=\beta_{0i}+\beta_{1i}\left(FS_{ti}-PMFS_{i}\right)+\beta_{2i}Wave_{ti}+e_{ti}$$

(2) $$\beta_{0i}=\gamma_{00}+\gamma_{01}PMFS_{i}+w_{0i}$$

(3) $$\beta_{1i}=\gamma_{10}+w_{1i}$$

(4) $$\beta_{2i}=\gamma_{20}$$

Equation (1) is the repeated-observation model, in which loneliness for person *i* at time *t* is modeled as a result of within-person variation in financial strain at the same time-point, and *e*_*ti*_ represents the random within-person error in loneliness at that time-point. In equation (1), *β*_1*i*_ indicates the expected difference in loneliness when financial strain at time *t* is one unit more than the mean for individual *i*. The variable Wave_*ti*_ in equation (1) is coded 0 = 2021, 1 = 2022, with *β*_2*i*_ indicating the expected difference in loneliness between waves; inclusion of wave serves the purpose of “de-trending” and ensures that the focal time-varying associations are not attributable to broader trends occurring over the time period under observation (Wang & Maxwell, 2015). The coefficient *β*_0*i*_ represents the between-person component of loneliness for individual *i*. Variation in between-person loneliness is then modeled in equation (2) by taking *β*_0*i*_ as the result of the mean level of financial strain across time-points for the same individual. Within equation (2), *γ*_01*i*_ indicates the expected difference in loneliness between individuals across waves at one unit higher for mean level of financial strain. Further, *w*_0*i*_ indicates residual random variation in the intercept across respondents. Equation (3) shows that the model includes a “random effect” *w*_1*i*_ for the slope *β*_1*i*_, which permits estimation of the degree to which the association between within-person financial strain and loneliness varies across individuals. Equation (4) indicates that a random effect for *β*_2*i*_ is not included in the model because inter-individual differences in trends and explanators of these differences are not germane to the current research. Not shown in this set of equations are the covariates, with the time-varying covariates entered into equation (1) and time-stable covariates entered into equation (2).^6^ A subsequent Wald test can be used to test the equality of *β*_1*i*_ and *γ*_01*i*_, thereby showing whether the consequences of between- and within-person financial strain for loneliness are significantly different (Allison, 2009).

Mattering and self-esteem can also be integrated into the within-between model. Person-mean-centered mattering and self-esteem are entered into equation (1) and person-mean mattering and self-esteem are entered into equation (2). Subsequently, interactions are used to test whether mattering and self-esteem shape the association between financial strain and loneliness. For mattering, three interactions are tested. First is the test of between-person moderation, in which person-mean mattering and person-mean financial strain are interacted to test whether the association between inter-individual financial strain and loneliness differs by between-person levels of mattering. The second interaction also involves between-person mattering, but is a cross-level interaction, in which person-mean mattering and person-mean-centered financial strain are interacted to test whether inter-individual mattering modifies the association between intra-individual financial strain and loneliness. The third interaction is the test of within-person moderation, in which person-mean-centered mattering and person-mean-centered financial strain are interacted to test whether intra-individual mattering modifies the association between intra-individual financial strain and loneliness. A similar set of interactions with self-esteem instead of mattering can be used to test the moderating role of self-esteem.

Mixed models typically accommodate respondent attrition by retaining respondents for all waves in which they participated (Bierman, 2014), but because means in measures across time and time-specific divergences from these means were of central interest, analyses were restricted to respondents who participated in both waves of the survey. An ancillary logistic regression model that included the baseline focal measures and a diverse set of additional characteristics indicated that less than 5% of the variance in attrition was explained by the full set of these factors, suggesting that between-wave attrition was largely completely at random, with the result that there was little bias in analyses due to attrition (Enders, 2010). To address possible attrition biases, though, a stabilized inverse propensity weight was created to adjust for attrition (Weuve et al., 2012), and then integrated into the original sampling weight by multiplication, with this final combination weight used in all analyses and applied at the person level in the mixed models. The final analytic sample with listwise deletion is 2384; as this is less than a 2% reduction, bias due to listwise deletion is minimal.

## Results

Before proceeding to the focal analyses, we note some important descriptive statistics. First, we present the bivariate correlations among our key constructs of mattering, self-esteem, and loneliness at baseline.^7^ Loneliness is moderately correlated with mattering (*r* = −.385) and self-esteem (*r* = −.439), and mattering and self-esteem also moderately correlated (*r* = .565), and all significant at *p* < .001. Ancillary mixed-effects models also examined whether a measure of wave—and therefore change over time—significantly predicted financial strain, mattering, self-esteem, and loneliness. These ancillary analyses showed that mean levels of financial strain significantly increased (*b* = .120, *p* < .001) and mean levels of mattering significantly decreased (*b* = −.019, *p* < .05). The mean change in self-esteem was not statistically significant (*b* = −.015), but there was also significant inter-individual variation in change in each of these measures (*p* < .001). Not surprisingly, then, there was also notable intra-individual variation in financial strain, mattering, and self-esteem, with additional analyses of intra-class correlation coefficients showing about 25% of variation in each measure was attributable to time-varying factors, indicating a substantial amount of temporal variation for only a one-year interval. There was also a significant decrease in loneliness between waves (*b* = −.040, *p* < .01), which likely reflected the period of this study in which social restrictions due to the COVID-19 pandemic were loosening. There was also significant variation between individuals in change in loneliness (*p* < .001), and about a third of variation in loneliness was attributable to time-varying factors. Variation in loneliness was therefore attributable to both between and within-person influences, and it is this variation that we next account for using the measures of financial strain, mattering, and self-esteem.

### Within-Between Analyses of Loneliness

Table 1 displays the results of the primary analyses. Model 1 shows a mixed-effects model of the bivariate association between financial strain and loneliness. Model 1 takes repeated observations of the same individuals into account, but does not separate financial strain into inter-individual and intra-individual components. In Model 1, financial strain is significantly and positively associated with loneliness. Based on these analyses, we would conclude that higher levels of financial strain are associated with greater loneliness when background controls are not taken into account.

**Table 1.** Within-Between Models of Financial Strain and Loneliness.

	Model 1			Model 2			Model 3			Model 4			Model 5			Model 6		
	Coef.	SE	*p*	Coef.	SE	*p*	Coef.	SE	*p*	Coef.	SE	*p*	Coef.	SE	*p*	Coef.	SE	*p*
Focal Predictors																		
Financial strain--not decomposed	.199	.020	***															
Inter-individual financial strain				.340	.025	***	.183	.024	***	.616	.111		.415	.103	***	.600	.115	***
Intra-individual financial strain				.048	.030		.036	.030		.076	.185	***	.412	.173	*	.260	.197	
Inter-individual mattering							−.162	.034	***	.070	.063		−.155	.035	***	.087	.079	
Intra-individual mattering							−.135	.035	***	−.135	.035	***	−.135	.035	***	−.136	.034	***
Inter-individual self-esteem							−.365	.029	***	−.362	.029	***	−.254	.052	***	−.386	.068	***
Intra-individual self-esteem							−.159	.034	***	−.159	.034	***	−.157	.034	***	−.157	.034	***
Interactions																		
Inter-individual mattering × inter-individual financial strain										−.125	.031	***				−.136	.043	**
Inter-individual mattering × intra-individual financial strain										−.011	.049					.104	.063	
Intra-individual mattering × intra-individual financial strain										−.331	.220					−.341	.240	
Inter-individual self-esteem × inter-individual financial strain													−.063	.027	*	.014	.039	
Inter-individual self-esteem × intra-individual financial strain													−.099	.043	*	−.157	.055	**
Intra-individual self-esteem × intra-individual financial strain													−.079	.203		.030	.221	
Time-Stable Covariates																		
Post-high school^a^							.069	.044		.068	.044		.069	.044		.067	.044	
Trade school^a^							.100	.045	*	.090	.045	*	.097	.045	*	.089	.045	*
University degree^a^							.092	.039	*	.087	.039	*	.090	.039	*	.086	.039	*
Age							.011	.003	***	.012	.003	***	.012	.003	***	.012	.003	***
Gender (Women = 1)							.129	.026	***	.133	.026	***	.132	.026	***	.132	.026	***
Visible minority							.049	.059		.046	.061		.048	.061		.046	.061	
Time-Varying Covariates																		
$25,000 to less than $50,000^b^							.148	.062	*	.155	.063	*	.147	.063	*	.156	.063	*
$50,000 to less than $100,000^b^							.170	.062	**	.171	.063	**	.169	.063	**	.173	.063	**
$100,000 to less than $150,000^b^							.155	.065	*	.149	.066	*	.151	.065	*	.153	.065	*
$150,000 and over^b^							.169	.071	*	.156	.071	*	.163	.071	*	.158	.071	*
Missing income^b^							.262	.070	***	.261	.071	***	.260	.071	***	.263	.071	***
Retired							−.030	.028		−.028	.027		−.032	.028		−.031	.027	
No partner							.209	.036	***	.212	.035	***	.209	.036	***	.212	.035	***
Number of people in household							−.053	.021	*	−.053	.020	**	−.056	.021	**	−.054	.020	**
In-person social contact							−.067	.010	***	−.067	.010	***	−.068	.010	***	−.067	.010	***
Electronic social contact							−.014	.011		−.013	.011		−.014	.011		−.013	.011	
Wave (2022 = 1)							−.045	.014	**	−.045	.014	**	−.043	.014	**	−.043	.014	**
Constant	1.214	.034	***	.987	.042	***	2.558	.259	***	1.708	.313	***	2.105	.299	***	1.739	.311	***
Log pseudolikelihood	−4775.656			−4765.905			−4271.788			−4256.613			−4263.975			−4251.505		
RandomEffects																		
*Slope of Within-Person Financial Strain (w*_*1i*_*)*	.068	.018	***	.165	.059	***	.142	.054	**	.142	.054	**	.142	.052	**	.138	.051	**
Between-person loneliness (w_0i_)	.318	.082	***	.381	.020	***	.245	.014	***	.241	.014	***	.244	.014	***	.241	.014	***
Covariance of w1i and w_0i_	−.049	.039		.012	.017		−.005	.014		−.005	.014		−.006	.014		−.005	.014	
*Within-Person Loneliness (e*_*ti*_*)*	.201	.012	***	.181	.012	***	.172	.011	***	.172	.011	***	.172	.011	***	.172	.011	***

^a^High school degree is reference.

^b^Less than $25,000 is reference.

*p ≤ .05. **p ≤ .01. ***p ≤ .001. *N* = 2384.

However, Model 2 differentiates between time-stable and time-varying financial strain. In Model 2, between-person financial strain is significantly associated with greater levels of loneliness, but the association between within-person financial strain and loneliness is not significant. Moreover, an ancillary Wald test showed that the coefficients for inter- and intra-individual components of financial strain are significantly different (*p* < .001). Disentangling inter- and intra-individual influences of financial strain on loneliness therefore reveals that inter- and intra-individual financial strain have distinct connections with loneliness in later-life. Furthermore, although a non-significant coefficient for intra-individual financial strain appears to indicate that within-person increases in financial strain are not associated with increases in loneliness, the random effect for time-varying financial strain is statistically significant (*p* < .001).^8^ This random effect shows that there are significant differences between individuals in the degree to which within-person variation in financial strain is associated with loneliness. We examine whether mattering and self-esteem contribute to explaining these between-person differences using cross-level interactions in subsequent models.

Model 3 includes background controls, along with the measures of mattering and self-esteem that are separated into time-stable and time-varying components. The association between time-stable financial strain and loneliness is reduced approximately 45% from Model 2. Ancillary analyses showed an approximately 15% of this reduction was due to the background controls, with an additional 30% due to mattering and self-esteem. The majority of the association between time-stable financial strain and loneliness is therefore robust to controls for mattering and self-esteem. Furthermore, Model 3 shows that mattering and self-esteem importantly offset the association between financial strain and loneliness, as the inter- and intra-individual aspects of mattering and self-esteem are significantly associated with lower levels of loneliness. The within-person associations are especially notable because these within person associations are purged of all time-stable influences and are therefore a form of econometric “fixed-effects models” that holistically control for *all* time-stable sources of confounding (Allison, 2009; Bierman et al., 2018). That mattering and self-esteem are both significantly associated with loneliness even with this extensive level of statistical control shows that both mattering and self-esteem have robust inverse associations with loneliness in later-life. Additionally, although not as robust to time-stable confounders, the between-person associations are still of interest because they indicate that the degree to which older adults continue to feel lonely over time can be independently explained by low levels of mattering and self-esteem. Irrespective of the buffering capabilities of mattering and self-esteem, then, these direct associations show that mattering and self-esteem can each independently serve to deter loneliness in later-life, thereby offsetting the degree to which financial strain foments loneliness.

The next set of models examines whether mattering and self-esteem modify the associations between financial strain and loneliness. Model 4 shows interactions with mattering. There is a significant interaction between inter-individual mattering and inter-individual financial strain, which indicates that between-person mattering shapes how between-person financial strain is associated with loneliness. The interaction between inter-individual mattering and intra-individual financial strain is not significant, nor is the interaction between intra-individual mattering and intra-individual financial strain.^9^ These non-significant interactions indicate that neither between-person mattering nor within-person mattering shapes how within-person financial strain is associated with loneliness.

**Figure 2.**
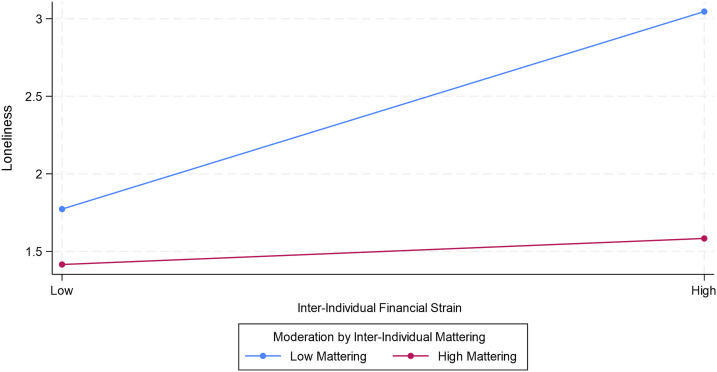
Consequences of inter-individual financial strain for loneliness.

To explicate the significant interaction in Model 4, Figure 2 plots the marginal association between inter-individual financial strain and loneliness at lower and higher levels of between-person mattering based on an ancillary model that includes only the interaction between inter-individual mattering and inter-individual financial strain.^10^ Figure 2 shows that the association between inter-individual financial strain and loneliness is positive at low levels of between-person mattering (*b* = .424, *p* < .001), with this association much weaker at higher levels of between-person mattering (*b* = .056, *p* > .10). A high degree of between-person feelings of social worth therefore negates the association between inter-individual financial strain and loneliness.

**Figure 3.**
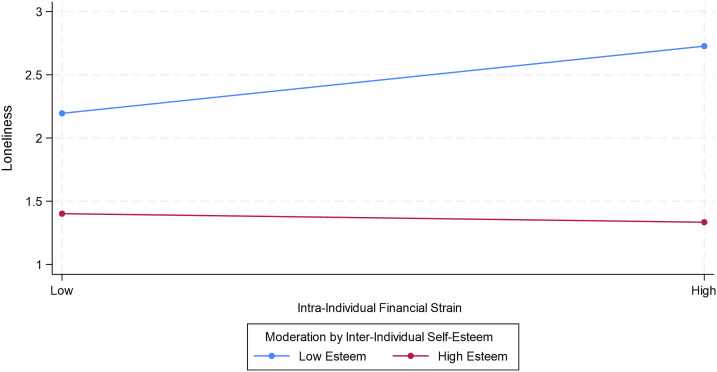
Consequences of intra-individual financial strain for loneliness.

Model 5 repeats the interactions tested in Model 4, but now using self-esteem instead of mattering. Model 5 shows a significant negative interaction between inter-individual self-esteem and time-stable financial strain. Because this interaction is negative, it is similar in form to Figure 2, in which higher between-person self-esteem weakens the association between inter-individual financial strain and loneliness. Additionally, an interaction between inter-individual self-esteem and within-person financial strain is significant in Model 5, indicating that between-person self-esteem modifies the association between intra-individual financial strain and loneliness. Figure 3 clarifies the meaning of this interaction by plotting the marginal association between intra-individual financial strain and loneliness at lower and high levels of between-person self-esteem. Figure 3 shows that within-person financial strain is associated with greater loneliness at lower levels of between-person self-esteem (*b* = .266, *p* < .05), but not at higher levels of between-person self-esteem (−.034, *p* > .10). Despite a non-significant overall association between intra-individual financial strain and loneliness, then, subsequent buffering analysis shows that there is a positive association between intra-individual financial strain and loneliness—but only in the context of low inter-individual self-esteem. However, Model 5 also shows that the interaction between intra-individual self-esteem and intra-individual financial strain is not significant, thereby indicating that the moderating role of self-esteem is restricted to its inter-individual aspects.

Model 6 tests the focal interactions from Models 4 and 5 together in one model, which serves to demonstrate the extent to which mattering and self-esteem provide independent moderating effects. There is only one substantial difference between the results in Model 6 and those shown in Models 4 and 5: In Model 6, the coefficient for the interaction between inter-individual self-esteem and inter-individual financial strain is substantially reduced and is no longer statistically significant. The appearance that between-person self-esteem shapes the association between inter-individual financial strain and loneliness is entirely attributable to the overlap between mattering and self-esteem. Moreover, comparison of the remaining significant interactions shows distinct roles for mattering and self-esteem: Higher levels of between-person mattering weaken the association between inter-individual financial strain and loneliness, whereas higher levels of between-person self-esteem weaken the consequences of the intra-individual aspect of financial strain for loneliness.^11^

## Discussion

Our analyses indicate that financial strain is associated with loneliness in later-life, but also demonstrate an important qualification to this association. When buffering by mattering and self-esteem is *not* taken into account, we find that variation between individuals in financial strain is associated with higher levels of loneliness, but variation within individuals in financial strain is not. Financial strain in later-life is the result of a process of increasing disparities across people’s lives (Dannefer, 2020; O’Rand, 2006; Pudrovska et al., 2005). That differences between older adults in financial strain are then associated with loneliness shows how loneliness in later-life is arranged in a hierarchy due to life-course processes of financial differentiation. The broader life-course financial context of loneliness is especially a concern because research suggests that individuals are approaching later-life increasingly saddled with economic burdens (Bierman, 2021). As a result, population aging will continue to be accompanied by increasing loneliness. Since loneliness is associated with a diverse set of negative health outcomes (Berg-Weger & Morley, 2020), greater financial supports that help to prevent difficulty affording necessities among older adults will be useful for not only deterring loneliness in older adults, but also strains on a healthcare system that is likely to already be stretched due to the growing population of older adults.

One objection to this pattern of findings may be that two waves of observations was not sufficient to detect the effects of financial strain. However, financial strain did change significantly between waves. Moreover, both mattering and self-esteem were similar in the degree to which their variation was attributable to within-person variation, and both significantly predicted loneliness. This pattern of findings emphasize that a lack of significance was not due to restricted variance, but instead a weak overall association. Most importantly, the association between intra-individual financial strain and loneliness was significant when stress buffering was taken into account. Thus, it does appear that there was sufficient variation in financial strain to detect an association at the intra-individual level, and it was the presence of stress buffering that concealed this association.

This research further shows that the self-concept provides a critical context for the consequences of financial strain for loneliness. The self-concept achieves this context in part by offsetting the consequences of financial strain for loneliness. At both the between- and within-person levels, greater levels of mattering and self-esteem were associated with lower levels of loneliness. The within-person associations are especially critical because they suggest that, even in the midst of increasing financial pressures, older adults can mitigate increases in loneliness by building a stronger sense of mattering and self-esteem. Although reinforcing these aspects of the self-concept may be difficult on an individual basis, sociological theory emphasizes that the self-concept is strengthened through individuals’ experiences of affirmatory social interactions (Cast & Stets, 2016). Interventions which seek to bolster older adults’ positive social engagement through community and volunteering activities are likely to provide a vital source of social significance and self-worth which will in turn offset loneliness caused by financial pressures. These programs are likely to be especially critical during times of societal-wide economic problems, such as increasing inflation, that may widely impact older adults (Bierman, Upenieks & Lee, 2023).

Mattering and self-esteem also formed an important context for the consequences of financial strain by serving as stress buffers. Buffering by each was, however, specific to inter- or intra-individual associations between financial strain and loneliness. Between-person mattering buffered the association between inter-individual financial strain and loneliness. Research on mattering shows that a sense of social significance results from not only strong and high quality social relationships, but also advantaged placement in structures of social stratification (Bonhag & Froese, 2022; Schieman et al., 2010). Advantaged levels of between-person mattering in later-life is therefore the result of the accumulation of advantages in social relationships and statuses across the life-course. That mattering is then protective of the effects of financial disadvantages also accrued throughout the life-course illustrates how loneliness in later-life is in part the consequence of a form of double-jeopardy: older adults most vulnerable to high levels of financial limitations that structure greater loneliness will also tend to have a lower reservoir of a psychological resource that would negate these effects. Greater attention should therefore be given to the way that both mattering and financial strain develop over the life-course to create disadvantages in loneliness in later-life, especially in terms of the ways that these developments can be interrupted as a means of preventing later-life loneliness.

Self-esteem constituted a form of buffering that was distinct from buffering by mattering. Inter-individual self-esteem acted in a cross-level form of moderation to prevent the consequences of intra-individual variation in financial strain for loneliness. In fact, it was only at low levels of between-person self-esteem that a significant association between intra-individual financial strain and loneliness was observed. This research therefore suggests that increases in financial strain associated with aging may indeed contribute to increases in later-life loneliness—but primarily for older adults who are lacking in self-esteem. As self-esteem is also based within pre-existing power structures (Cast & Stets, 2016), this research shows how increases in financial strain during later-life are likely to be especially critical for loneliness among disadvantaged older adults. More broadly, this pattern of findings underscores the utility of applying a stress process perspective to the study of loneliness in later-life. The consequences of increases in stressors on loneliness must be understood as inherently shaped by buffering resources, and ignoring this buffering context may understate the extremity of the effects of these stressors for disadvantaged older adults.

It is less clear why mattering buffered the time-stable aspects of financial strain, but self-esteem buffered the time-varying aspects of financial strain. This could be due to the specific aspects of worth that each component of the self-concept represents. As an indicator of a sense of self-worth, self-esteem may be especially important in reassuring older adults as they experience increases in financial stress that they are still good people with redeeming qualities. Conversely, mattering may be especially useful in weakening a sense that one is less than another due to greater relative disadvantages in financial problems, which bolsters social engagement and the quality of social relationships. Additional research should, however, more closely examine the mechanisms through which both mattering and self-esteem prevent effects of financial disadvantages on loneliness to better understand these buffering effects.

It is also important to note that the buffering role of self-esteem was overstated when buffering by mattering was not taken into account. In a model that tested only buffering by self-esteem, between-person self-esteem also appeared to buffer the effects of inter-individual financial strain; inclusion of buffering effects by mattering negated this interaction, though. Although there is an extensive body of research on self-esteem (Ziegler-Hill, 2013), much less research has been conducted on mattering, especially the buffering effects of mattering. Both focus on an individual’s sense of worth, though, with the result that the appearance of the protective effects of self-esteem may represent buffering by mattering when mattering is not included in analyses. Additional research on stress buffering in later-life should, therefore, more commonly jointly consider both mattering and self-esteem for a more accurate depiction of the buffering effects by each.

It should further be noted that neither within-person self-esteem nor within-person mattering provided buffering effects. This pattern of findings suggests that the consequences of financial strain for loneliness in later-life are substantial enough that increasing one’s sense of worth cannot deter these effects. Yet, even if increases in these aspects of the self-concept do not weaken the effects of increasing levels of financial strain, strengthening mattering and self-esteem in later-life may be critical by offsetting the effects of both intra- and inter-individual financial strain for loneliness. This research therefore shows how delineating the between and within-person aspects of psychological resources is useful for specifying the specific ways that these resources can deter loneliness in later-life. In the case of financial strain, the between-person aspects of mattering and self-esteem provide buffering effects, while both the between- and within-person aspects of these components of the self-concept provide offsetting effects.

Several weaknesses to this research should also be addressed. First, the degree to which between-person financial strain was associated with loneliness was reduced 30% with controls for mattering and self-esteem, suggesting that these components of the self-concept are likely to partially mediate this association. We did not examine this question further because mediation was not a focus of this study, but also because a full test of mediation would require clearer attention to the role that reductions in social activities and quality of social bonds may play in these processes. More fully testing mediators in the association between financial strain and loneliness—while also attending to between-person and within-person levels of analysis—is therefore an important direction for future research. Second, repeated observations over a longer period of time would help to produce a more reliable estimate of inter-individual influences. Third, this research could not take into account nations which have higher levels of collectivism that in North America, as well as more developed economic support systems for older adults. Both factors may weaken the consequences of financial strain for loneliness, and in turn obviate the need for buffering by the self-concept. Additional cross-national research should therefore consider these macro-level contingencies in greater depth.

### Conclusion

Identifying the factors that lead to loneliness in later-life is an especially important area of study because aging is associated with a greater risk of loneliness, which in turn poses a threat to the well-being of older adults. This research suggests that financial strain may be a key mechanism through which older adults are more at risk of loneliness. Financial strain may play this role both because more recent cohorts of individuals are entering later-life with financial burdens, and also because financial strain tends to increase in later-life. Maintaining a strong sense of worth can help to both prevent and offset these effects, though. Even if practitioners cannot prevent financial strain in older adults, working to create programs that bolster self-esteem and mattering is likely to be key for thwarting loneliness in later-life.

## Notes

1. A fourth form of buffering—in which intra-individual self-esteem and mattering weaken the effects of between-person financial strain—is much less plausible because this would suggest that a time-consistent influence is modified by a time-variant factor. Additionally, ancillary analyses showed no evidence of this form of buffering. We therefore do not address this form of buffering in the main focus of the paper.

2. When weighted, at baseline, the sample’s mean age was 72.3, with 52.8% women, 14.6% having a high school degree and 39% a university degree, 6.2% identifying as a visible minority, 84.5% retired, and 37.3% not living with a romantic partner.

3. The response format for mattering was employed to keep consistent with the response format for additional components of the self-concept, but this 1-5 response format did deviate from a more common response 1-4 response format used in this scale (ranging from “not at all” to “a lot”). As pointed out by a reviewer, the acceptable internal consistency of the scale in the current study suggests that this measurement variation did not appreciably change the measure as whole, as do the findings in the current study which concur with previous findings linking low mattering with loneliness (e.g., Flett et al., 2016).

4. Age was not measured as time-varying because the study of age-related changes would most appropriately require observation of broader swaths of time over multiple time-points (Bierman, 2014), and general changes over time were addressed by “de-trending” the analyses through the inclusion of a measure of wave, as described in the plan of analysis.

5. Preliminary analyses indicated that between a quarter and a third of the variance in the focal measures was attributable to within-person variability. As this is similar to methodological demonstrations of the analysis of intra- and inter-individual influences in mixed models (e.g., Hoffman & Stawski, 2009), it is considered sufficient for the current analyses.

6. Also not shown in the set of equations, but included in all analyses, is a covariance term between the between-person random effects *w*_0*i*_ and *w*_1*i*_.

7. When weighted, the mean for loneliness at wave 1 was 1.577 (SD = .815) and at wave 2 was 1.536 (SD = .777). The mean for financial strain at wave 1 was 1.613 (SD = .735) and at wave 2 was 1.734 (SD = .801). The mean for mattering at wave 1 was 3.662 (SD = .614) and wave 2 was 3.643 (SD = .612). The mean for self-esteem at wave 1 was 3.992 (SD = .708) and at wave 2 was 3.977 (SD = .700). A fuller analyses of changes in financial strain, mattering, and self-esteem—along with demographic predictors of these changes—can be found in Bierman et al. (2023).

8. Test of the random effect for a slope is based on a one-tailed test, with a one-tailed test required for accurate testing because the random effect of the slope cannot be negative (LaHuis & Ferguson, 2009). By extension, the tests of the random effects for within- and between-person loneliness are also one-tailed.

9. Ancillary analyses that tested these interactions individually did not show substantively different results, as did ancillary analyses individually testing interactions with self-esteem.

10. In Figure 2, values of 1.5 and 4.5 are used as the respective lower and higher values of between-person mattering and financial strain, with these values chosen because they represent lower and higher ends without capitalizing on the most extreme values. In Figure 3, the same values are used for lower and higher levels of inter-individual self-esteem. Because intra-individual financial strain is a measure of variation within individuals over time, its range is more restricted, and Figure 3 therefore uses values of −1 and positive 1 as the respective values of low and high levels of intra-individual financial strain.

11. An ancillary model that retained only the significant interactions in Model 6 also showed the same pattern of findings.
